# Supplementary material for: MicroRNAs with Multiple Targets of Immune Checkpoints, as a Potential Sensitizer for Immune Checkpoint Inhibitors in Breast Cancer Treatment
Source: Cancers (Basel). 2023 Jan 29;15(3):824. doi: 10.3390/cancers15030824 (PMC9913694; doi:10.3390/cancers15030824)
Supplement: Supplementary file 1 [file cancers-15-00824-s001.zip › cancers-2151733-supplementary.pdf]

miR-215-5p,miR-411-3p,miR-493-5p, miR-494-3p, miR-495-3p, miR-548j-5p and miR-93-3p: The expression of these seven miRNAs are different between responders with an overall survival(OS) > 6 months and the responders with an OS < 6 months in non-small cell lung cancer(NSCLC) patients treated by nivolumab, showing good predicting for ICIs therapy [1].

miR-6885-5p, miR-4698, and miR-128-2-5p: In esophageal squamous-cell carcinoma patients treated by nivolumab , the three miRNAs can distinguish responders from non-responders [2].

miR-222:the melanoma patients advancing on ICI therapy have greater expression levels of miR-222 than the patients who have a clinical benefit from immune checkpoint blockage [3].

miR-320d, miR-320c, miR-320b: In NSCLC patients, baseline levels of the three miRNAs are significantly lower in responders to ICI therapy compared to non-responders [4].

miR-208a-5p,miR-574-5p: NSCLC patients with expression levels of miR-208a-5p and miR-574-5p below the median have a significantly longer OS following the start of immune-checkpoint blocking than patients with expression levels above the median [5].

let-7,miR-125a,miR-99b,miR-146b,miR-125b: Melanoma patients with low expression level of the five miRNAs undergoing immune-checkpoint blockade with either ipilimumab or nivolumab displayed a statistically significantly better OS compared to high expression level patients [6].

Reference

1. Halvorsen, A.R.; Sandhu, V.; Sprauten, M.; Flote, V.G.; Kure, E.H.; Brustugun, O.T.; Helland, . Circulating microRNAs associated with prolonged overall survival in lung cancer patients treated with nivolumab. *Acta Oncol.* **2018**, *57*, 1225–1231. https://doi.org/10.1080/0284186x.2018.1465585.
2. Sudo, K.; Kato, K.; Matsuzaki, J.; Takizawa, S.; Aoki, Y.; Shoji, H.; Iwasa, S.; Honma, Y.; Takashima, A.; Sakamoto, H.; et al. Identification of serum microRNAs predicting the response of esophageal squamous-cell carcinoma to nivolumab. *Jpn. J. Clin. Oncol.* **2020**, *50*, 114–121. https://doi.org/10.1093/jjco/hyz146.
3. Galore-Haskel, G.; Nemlich, Y.; Greenberg, E.; Ashkenazi, S.; Hakim, M.; Itzhaki, O.; Shoshani, N.; Shapira-Fromer, R.; Ben-Ami, E.; Ofek, E.; et al. A novel immune resistance mechanism of melanoma cells controlled by the ADAR1 enzyme. *Oncotarget* **2015**, *6*, 28999–29015, doi:10.18632/oncotarget.4905.
4. Peng, X.X.; Yu, R.Y.; Wu, X.; Wu, S.Y.; Pi, C.; Chen, Z.H.; Zhang, X.C.; Gao, C.Y.; Shao, Y.; Liu, L.; et al. Correlation of plasma exosomal microRNAs with the efficacy of immunotherapy in EGFR/ALK wild-type advanced non-small cell lung cancer. *J. Immunother. Cancer* **2020**, *8*, doi:10.1136/jitc-2019-000376.
5. Genova, C.; Coco, S.; Rossi, G.; Longo, L.; Chiorino, G.; Ostano, P.; Guana, F.; Metro, G.; Baglivo, S.; Ludovini, V.; et al. An exosomal miRNA signature as predictor of benefit from immune checkpoint inhibitors in non-small cell lung cancer. *Ann. Oncol.* **2020**, *31*, S825–S826, doi:10.1016/j.annonc.2020.08.1591.
6. Huber, V.; Vallacchi, V.; Fleming, V.; Hu, X.Y.; Cova, A.; Dugo, M.; Shahaj, E.; Sulsenti, R.; Vergani, E.; Filipazzi, P.; et al. Tumor-derived microRNAs induce myeloid suppressor cells and predict immunotherapy resistance in melanoma. *J. Clin. Investig.* **2018**, *128*, 5505–5516, doi:10.1172/jci98060
